# Benefits of Pilates on Depression, Anxiety, and Stress: An Observational Study Comparing People Practicing Pilates to Non-Active Controls

**DOI:** 10.3390/healthcare13070772

**Published:** 2025-03-31

**Authors:** Sara Guidotti, Alice Fiduccia, Giulia Morisi, Carlo Pruneti

**Affiliations:** Clinical Psychology, Clinical Psychophysiology, and Clinical Neuropsychology Laboratory, Department of Medicine and Surgery, University of Parma, 43126 Parma, Italy; alice.fiduccia@unipr.it (A.F.); giulia.morisi@studenti.unipr.it (G.M.); carlo.pruneti@unipr.it (C.P.)

**Keywords:** anxiety, depression, lifestyle, pilates, stress, somatization

## Abstract

**Background**: The positive effects of Pilates on balance, resistance, strength, and flexibility have been described several times. Additionally, positive effects on psychological symptoms, such as anxiety and depression, were documented. However, the change in behaviors at risk for stress-related physical disorders has not yet been validated in a group of people practicing Pilates. In light of these assumptions, changes in risk behaviors for stress-related physical disorders were assessed along with psychological symptoms, comparing a group of people practicing Pilates with non-active controls. **Methods**: The study was observational. Data from an experimental group of twenty-five people practicing Pilates (five males and 20 females between 50 and 64 years old) were compared to those of a control group of 24 people (seven males and 17 females between 54 and 65 years). Psychological symptoms were assessed through the Symptom Questionnaire (SQ) while the P Stress Questionnaire (PSQ) investigated risk behaviors for stress-related physical disorders. **Results**: The analyses attested a significant decrease in anxiety (time × group effect: F = 17.38; *p* < 0.001), depression (time × group effect: F = 5.44; *p* < 0.05), and somatization (time × group effect: F = 11.25; *p* < 0.01), as well as an improvement in stress-risk behaviors, especially in the ability to benefit from spare time by “taking one’s mind away” from commitments (time × group effect: F = 8.56; *p* < 0.01). **Conclusions**: Although the literature describes benefits for anxiety and depression following moderate-intensity sporting activities, our study also noted positive effects from Pilates on stress and psychological symptoms. Our findings suggest that Pilates may be appropriate for people who can perform light to moderate exercise.

## 1. Introduction

The Ottawa guidelines serve as a useful reference for policymakers and health professionals regarding physical activity (PA) [1]. Increased life expectancy, reduced birth and infant mortality rates, and the increase in chronic diseases related to unhealthy lifestyles led to significant demographic changes and epidemiological events in recent decades. Addressing these medical, social, and financial challenges requires a fundamental review of health system strategies and the underlying conceptual model. Replacing the current biomedical and disease-focused approach with a more holistic approach aimed at the overall health of the individual is required. Citizens’ empowerment, active involvement, responsibility, and self-determination in maintaining and improving their health conditions are essential resources to achieve these goals [2,3].

Promoting efficient PA and considering an inactive lifestyle as a risk factor for chronic diseases, both physical and mental, could support new health policies [4]. The goal that the population should strive for should be to reach and maintain for as long as possible that state of well-being through PA programs [5].

Scientific literature identifies different types of PA that can be used to manage stress and various psychological symptoms associated with it [6]. Over the years, physical training manifested to be an effective strategy for improving people’s quality of life and emotional health [7,8]. Leading an active lifestyle through regular PA offers numerous health benefits for individuals of all ages. Moreover, studies indicated that PA can be a valuable tool in preventing and treating mental health disorders, such as anxiety and depression [9]. More specifically, it has been suggested that aerobic activities, such as walking, in addition to improving physical fitness, bring benefits to quality of life and mood disorders [10,11].

One form of aerobic activity is Pilates, whose objective is to assume correct posture and to give greater harmony and fluidity in movements. As the discipline is based on three basic principles: coordination, balance, and awareness, Pilates requires different types of exercise (i.e., balance, resistance, muscle strength, and flexibility) and attention to muscle control, posture, and breathing [12,13]. Pilates is believed to train both the body and the mind as it is at the service of psychophysical well-being and rehabilitative and preventive gymnastics [14]. Pilates involves low to moderate-intensity exercises that are easily learned, promoting an improvement in flexibility, dynamic balance, and muscular resistance in a short time [15].

Recent meta-analytic findings document moderate to large effects of supervised Pilates training on the physical well-being of clinical populations [16]. By way of illustration, Pilates was validated to be useful for reducing levels of depression, anxiety, and fatigue in patients with multiple sclerosis [17] and in female patients with breast cancer [18]. Furthermore, this practice resulted in a reduction in levels of depression, anxiety, and stress in overweight and obese individuals [6] as well as suffering from chronic non-specific low back pain [19], tension-type headache [20], and pediatric type 1 diabetes [21].

Nonetheless, only a few studies investigated the effects of Pilates on the mental health outcomes of subjects without physical disorders [16], mainly focusing on depression [22,23] and, to a lesser extent, on anxiety [11]. Conflicting results were described for anxiety, whereas a systematic review did not describe the same positive effects found for depression [24]. Specifically, a couple of studies analyzed the effectiveness of Pilates exercises in reducing mood symptoms in healthy women [23] and men [25]. Preliminary results on a healthy population involved university students, validating Pilates’ efficiency on depression status, pain, functionality, and quality of life [26]. Since the benefits of Pilates have been studied mainly concerning organic pathologies [16] or depression [22], the current literature highlights the need to carry out further studies to confirm the beneficial effect of Pilates on other outcomes of mental health, such as stress and anxiety. To our knowledge, there is only a study by Janssen et al. [27], which found Pilates as a potential stress-reducing intervention in pregnancy.

However, it is crucial to highlight the gap in knowledge about Pilates, as it has not been studied sufficiently in non-clinical populations.

In light of these hypotheses, the present pilot study aimed to preliminarily explore the effectiveness of Pilates practice through an observational study. Specifically, it was hypothesized to confirm a significant difference between pre-post training symptoms of depression and anxiety in a group of people practicing Pilates once a week for three months compared to a group of inactive controls. Nonetheless, the main objective of the research was to analyze the pre-post changes in somatization and risk behaviors for stress-related physical disorders following the Pilates program.

## 2. Materials and Methods

### 2.1. Participants

The study was an observational study that followed the Transparent Reporting of Evaluations with Non-randomized Designs (TREND) Reporting Guidelines for Non-randomized/Quasi-Experimental Study Designs.

For the experimental group (i.e., people practicing Pilates), inclusion criteria were being between 50 and 65 years of age; having a valid medical certificate to engage in non-competitive PA; being engaged in a weekly Pilates program for the first time; having no history of psychiatric or neurological syndromes (i.e., previous head trauma, epilepsy, etc.) or physical conditions (i.e., sensory disturbances of vision and hearing) that could limit the administration of the tests; and having completed informed consent.

For the control group (i.e., non-active people), inclusion criteria were being between 50 and 65 years of age; being inactive (not engaged in a PA program); having no history of psychiatric or neurological syndromes (i.e., previous head trauma, epilepsy, etc.) or physical conditions (i.e., sensory disturbances of vision and hearing) that could limit the administration of the tests; and having completed informed consent.

### 2.2. Procedure

Participants were consecutively recruited in the Clinical Psychology, Clinical Psychophysiology, and Clinical Neurophysiology Laboratories of the Department of Medicine and Surgery of the University of Parma. The researchers provided information about the purpose of the study before administering the questionnaires.

The experimental procedures were completed with the 1964 Declaration of Helsinki of the World Medical Association and the 2005 Universal Declaration on Bioethics and Human Rights of UNESCO.

### 2.3. Measures

Psychological symptoms were investigated through the Symptom Questionnaire [28]. The SQ is a self-assessment questionnaire composed of 92 items grouped into four scales, namely Anxiety (A), Depression (D), Somatization (S), and Hostility (H). The clinical cut-off corresponds to four for all the scales of the test. The SQ has been shown to have excellent test-retest reliability [29] with high levels of sensitivity and specificity (80% and 76% in general medicine, 86% and 74% in hospital medicine departments, and 83% and 85% in emergency departments, respectively) [30]. This test has weekly, daily, and hourly versions. In this study, the first version was used for the pre-post investigation.

Risk behavior for stress-related physical disorders was assessed through the P Stress Questionnaire [31], an instrument composed of 32 items, grouped into six scales: Sense of Responsibility (SR; includes attitudes related to taking life and personal duties too seriously), Vigor (V; includes items referring to the feeling of having characteristics, such as vitality, energy and resistance to stress), Stress Disorders (SD; consists of items related to problems and difficulties usually related to stress reactions, such as lack of sexual interest and difficulty falling asleep), Precision and Punctuality (PP; includes behaviors characterized by spite, precision, and punctuality), Leisure Time (ST; is related to items regarding self-care and the ability and possibility to relax and take breaks from work) and Hyperactivity (H; refers to behaviors characterized by extreme activity and the presumption of being able to resist fatigue well). Raw scores are converted into standardized scores on a scale from 1 to 9, with a mean of 5 and a standard deviation of 1.96. Cronbach’s alpha calculated, including all scales, was 0.76.

### 2.4. Pilates Training

The Pilates method proposed to the study group employed a series of basic exercises performed free body on a mat. Each exercise can be adapted to make it easier for the client or to increase the challenge. During each exercise, special emphasis is placed on correct breathing. The importance of maintaining and achieving axial lengthening, strengthening core control, correct organization of the shoulder girdle, mobilizing the spine and aligning the upper and lower limbs, and integrating the entire body is underlined. The study group participated in a Pilates Matwork teaching for three months, attending the gym once a week for an hour of lessons. Every week, the operator proposed different exercises and sequences, making small changes to ensure physical and mental benefits already at the end of the lesson. The goal was to take them on an introspective journey through the practice of Pilates. To make the matwork exercises more accessible or challenging, small tools were used, including the magic circle and softball, which the study group used during the sessions. For instance, the magic circle represents a ring made of a semi-flexible material, with a diameter of approximately 30–40 cm, equipped with side handles complete with bearings on each side. This tool is extremely advantageous for strengthening the entire body and training muscles without putting excessive stress on the joints. Its versatility allows it to be used with both the upper and lower limbs. The circle allows the performing of exercises characterized by both isometric and isotonic movements. In other words, during isotonic movement, the muscle shortens by moving a constant weight for the duration of the contraction. In contrast, isometric contraction implies that the muscle contracts without changing its length, opposing an immobile resistance. According to one of the manuals on the subject, this tool is useful for many reasons. To illustrate, it improves the body’s elasticity and makes all the muscles work, allows it to develop greater concentration, corrects posture, and improves breathing. On top of this, it can be a great way to relax too. On the other hand, a softball, with a diameter of approximately 20 cm, is used to increase muscle load, concentration, greater resistance, and to obtain more conscious stability. It can be used to challenge balance and stability, thus, requiring a more conscious and profound activation of the muscles. It can be used to create asymmetry in the body and use deep musculature to keep the body aligned. A particular and very beneficial sequence for the cervical and lumbar area of the spine is the one in which the deflated ball is used and positioned under the sacrum or the head so that it wraps around the neck and supports the cervical spine. In both cases, the ball absorbs a part of the body weight and allows the relaxation and unloading of specific sensitive areas. Once removed, the softball in the affected areas generates a sensation of pleasure, relaxation, and well-being.

### 2.5. Statistical Analysis

SPSS (version 28.0.1.0; IBM Corp, Armonk, NY, USA) was used to conduct the planned statistical analyses. Differences between groups (people practicing Pilates vs. non-active controls) in socio-demographic (e.g., gender, age, education, employment level, and marital status) and clinical variables (e.g., psychological symptoms and risk behavior for stress-related physical disorders) at T0 were tested using chi-square tests and an independent-samples *t*-test.

Adherence to the assumptions for conducting parametric statistics allowed several repeated measures ANOVAs to be conducted on each SQ and PSQ scale to assess change over time (from T0 to T1) in psychological symptoms (i.e., SQ scales) and risk behavior for stress-related physical disorders (i.e., PSQ scales). Specifically, the time × group interaction effect was calculated by obtaining the F-value and the related *p*-value and associated with an effect size measure (η^2^).

## 3. Results

Assuming an effect size F equal to 0.25, a type I error of 5% (α = 0.05), and a type II error of 5% (β = 0.05; power = 95%), an a-priori power calculation using GPower 3.1, revealed that 54 participants were required. Even if the subjects recruited in the actual research were less than 54, a post hoc power analysis certified that a sufficient power of 0.93 was achieved with the current sample size of 49 for the conduct of the repeated measures ANOVA.

**Figure 1:**
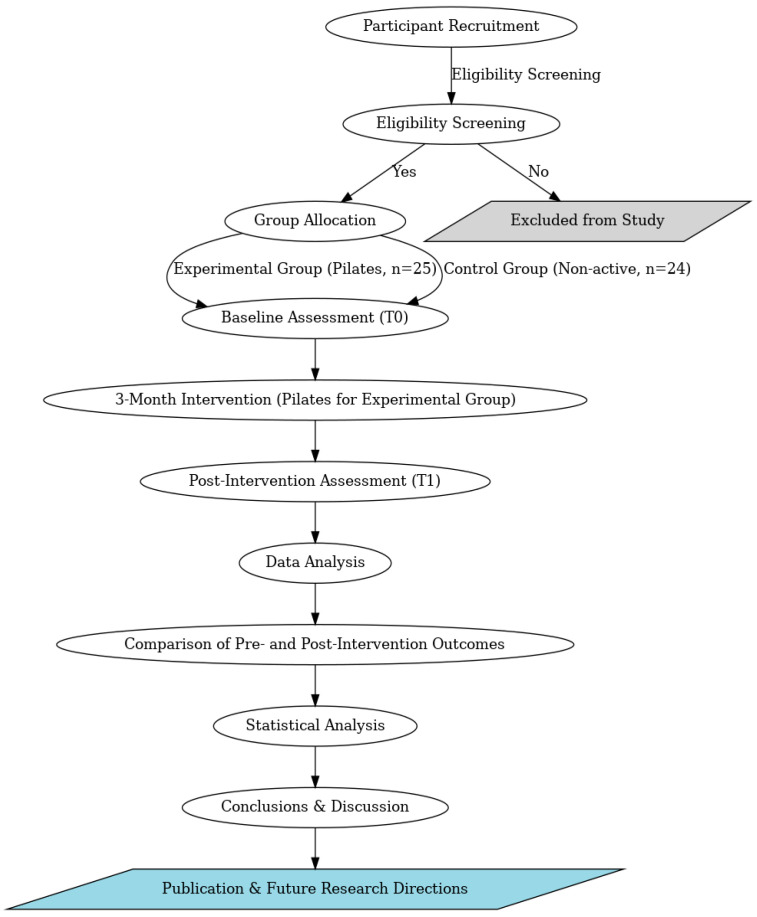
Flowchart of the study.

The actual research consecutively recruited and compared 25 people practicing Pilates to a group of 24 non-active controls (Figure 1).

Looking at the baseline comparison of the two groups, no significant differences were observed for the socio-demographic (Table 1) and clinical variables (Table 2) at T0 (pre-training).

Looking at the change over time in the two groups, a time × group interaction effect emerged in the anxiety, depression, and somatization scales of the SQ (Table 3) and in the stress disorders and spare time scales of the PSQ (Table 4).

Specifically, the time × group interaction of Anxiety scores was associated with a *p* < 0.001 and characterized by a partial eta squared indicative of a large effect (η^2^ > 0.14). The partial eta squared of the time × group interaction of somatization scores was also indicative of a large effect (η^2^ > 0.14), associated with a *p* < 0.01. Finally, the partial eta squared of the time × group interaction of depression scores was indicative of a medium effect (η^2^ > 0.06), with a *p* < 0.05.

Looking at the repeated measures ANOVA for the PSQ scales, the partial eta squared of the time × group interaction for Spare Time scores was indicative of a large effect (η^2^ > 0.14), associated with a *p* < 0.01. The partial eta squared of the time × group interaction for stress disorder scores was indicative of a medium effect (η^2^ > 0.06), with a *p* < 0.05.

## 4. Discussion

The main objective of this research study was to investigate the psychological effects of a Pilates program on a group of people who practiced it versus non-active controls. The study aimed to examine how specific Pilates exercises, implemented to achieve physical benefits, can improve mental health and, more specifically, psychological symptoms and risk behaviors for stress-related physical disorders.

As already mentioned, previous studies investigated the role of Pilates in reducing psychopathological symptoms, such as anxiety and depression, in clinical populations [16]. Several studies manifested that the practice of Pilates can lead to a significant reduction in anxiety, depression, and fatigue in female patients with breast cancer and people with multiple sclerosis [17,18]. Vancini and colleagues [6] found that practicing Pilates resulted in decreased levels of depression, anxiety, and stress in overweight and obese individuals. Furthermore, the positive effects of Pilates on mental well-being were observed in subjects suffering from chronic non-specific low back pain, tension-type headache, and pediatric type 1 diabetes [19,20,21].

Nevertheless, in our study, interesting results were found concerning the reduction in anxiety and depression in a non-clinical population, being in line with the limited evidence from healthy populations [22,23,26]. On top of this, our investigation found an improvement in anxiety symptoms, although the 2024 review by Dong and colleagues described conflicting findings across research groups [24]. One of the main points is the augmentation in the ability to attain free time as well. To our knowledge, our study is one of the few that described the evaluation of Pilates not only for anxiety and depression but, also, for stress-related physical disorders and associated risk behaviors.

The findings of our study confirm the effectiveness of Pilates in reducing depression and anxiety as well as somatization and risk behavior for stress-related disorders. However, a key aspect that deserves further exploration is the underlying mechanisms responsible for these benefits, particularly the role of breathing techniques, mindfulness, and neuromuscular engagement. More specifically, breathing is a fundamental component of Pilates and plays a crucial role in modulating autonomic nervous system (ANS) activity. Diaphragmatic and controlled breathing promotes greater cardiac coherence, positively influencing the balance between the sympathetic and parasympathetic branches of the ANS [32]. Previous studies demonstrated that conscious breathing techniques can reduce the hyperarousal of the ANS associated with chronic stress, modulating physiological responses to anxiety [33].

Furthermore, Pilates was described as a somatic practice that integrates elements of mindfulness [34]. During exercise execution, attention is directed toward movement quality, posture, and breathing, all of which foster enhanced body awareness “at the present moment”. In this regard, Mindfulness applied to PA was linked to reduced symptoms of anxiety and depression, as well as improved emotional regulation [35]. The controlled activation of deep musculature in Pilates contributes to reducing chronic muscle tension, a factor frequently associated with stress and anxiety disorders [36]. Regular Pilates practice improves proprioception and neuromuscular control, reducing stress reactivity through biomechanical and neurophysiological mechanisms [37].

Notwithstanding, our findings must be read in light of limitations. First, the small sample size and the imbalance in favor of women represent the major criticism found in previous studies [16]. Additionally, the control group was composed of inactive people, which is a limitation that future studies should overcome. Future research could involve control subjects who perform a kind of PA (i.e., walking) to better underline the specific positive effects attributable to Pilates. Furthermore, objective measurements (i.e., psychophysiological assessment) could be integrated into the efficacy evaluation to highlight the impact on stress management [38].

In light of the evidence on Pilates performed in the gym, the group effect cannot be neglected. The conduct of group Pilates is a transversal factor that favors the reduction of loneliness in group PA programs. Recent studies provided evidence to support that the benefits deriving from belonging to a social group also extend to belonging to sports and PA groups. In summary, studies found that membership in PA groups is associated with greater life satisfaction, happiness, and self-rated health [39,40]. This finding deserves careful consideration, especially in light of the consequences of the COVID-19 pandemic [41,42,43].

Despite these limitations, the possible clinical implications could be considerable, as involving groups of people with symptoms of anxiety, depression, and stress in Pilates programs could be effective. Our findings attested that significant mental health outcomes were achieved with a three-month weekly Pilates program, despite the World Health Organization guidelines [5] suggesting at least 150–300 min/week of Moderate aerobic PA for improving the psychophysical health of the population between 18 and 64 years. Thus, it is possible that the benefits of PA on psychological and social levels (i.e., reduction in isolation and improvement of socialization and mood) [44] and the psychobiological level (i.e., greater release of beta-endorphins in the nervous system capable of promoting physical and mental well-being) [45], as indicated by the WHO guidelines, can also be obtained from Pilates training, such as the one described in our study. Nevertheless, our findings corroborated the benefits of Pilates on stress management. Since stress vulnerability is considered a precursor of psychological and psychopathological symptoms (i.e., anxiety and depression) [46], certifying the effectiveness of Pilates in reducing them may represent a prevention tool. Nevertheless, Pilates could be exploited for the early intervention of psychological distress signals, even before vulnerability to stress evolves into anxiety and depression.

The relaxation obtained through Pilates can favor, promote, and maintain a state of relaxation for longer. The term relaxation, in contrast to that of stress, should be interpreted as an internal state of pleasantness and relaxation that favors the detachment of the mind from the problems (or rather, from the stressors) that life presents to everyone [47]. The etymology of the term itself refers to “liberation” and, therefore, to the achievement of a pleasant sensation of “letting go”. This should be the main meaning to be attributed to the term relaxation, intended not only as the relaxation of one or more muscle fibers but rather as the gradual learning of greater flexibility and elasticity, both muscular and mental. Pilates aims to teach people to be aware of themselves, their bodies, and their minds, and is understood as unique, dynamic, and functional entities in mutual interconnection. Pilates argued that: “physical fitness is the first criterion of happiness”. Therefore, encouraging healthy, PA through the conduct of efficient programs, such as Pilates, is a fundamental public health objective, as it can help to effectively reduce levels of stress, anxiety, and depression [48]. Given that Pilates is a promising non-pharmacological method for improving health, it is essential to significantly invest in its promotion [4].

## 5. Conclusions

The benefits associated with Pilates in promoting the improvement of the ability to release anxious tension and reduce the psychophysical response to stress have been told some time ago, as described by an anecdote. At the outbreak of the First World War, Pilates, the inventor of the discipline, was interned in a prison camp and maintained his commitment to training, also involving his fellow prisoners. It seems that those who continued the rigorous and effective Pilates training avoided contagion from the “Spanish flu”, which in that period (1918–1921) affected millions of victims throughout the world, with over 200,000 deaths in the UK alone.

Our research aimed to investigate the psychological effects of Pilates in a group of people who practiced it once a week. We wanted to examine the potential indirect effects of performing Pilates as a sporting activity on mental health and, specifically, on psychological symptoms and risky stress-related behaviors. Previous scientific literature already corroborated the positive effects of Pilates on physical health as well as depression and anxiety. Nevertheless, our study is the first to suggest the possible benefits of a weekly Pilates program on depression, anxiety, and stress. We found that engaging in Pilates might have a positive impact on the ability to take advantage of having some time to oneself and “take one’s mind away” from work commitments. Our results add to the existing literature by documenting how even a weekly Pilates protocol can promote the management of stress, anxiety, and depression. Health policies should be encouraged to implement non-pharmacological treatments for those people who are limited in performing PA at high intensity and frequency, bearing in mind the results of these types of studies.

## Figures and Tables

**Table 1:** Comparisons of socio-demographic characteristics between the Pilates group and the control group.

Variable	Pilates Group	Control Group	t or χ^2^	*p*
	(n = 25)	(n = 24)		
Age, M (SD)	57.52 (4.78)	59.67 (3.63)	t (48) = 1.77	0.1
Sex, N (%)			χ^2^ (1, N = 49) = 0.56	0.22
Male	5 (20%)	7 (29.17%)		
Female	20 (80%)	17 (70.53%)		
Marital status, N (%)			χ^2^ (2, N = 49) = 0.26	0.73
Unmarried	4 (16%)	3 (12.50%)		
Married/cohabitant	16 (64%)	17 (70.83%)		
Separated/Widowed	5 (20%)	4 (16.67%)		
Education Level, N (%)			χ^2^ (2, N = 49) = 0.01	0.62
Middle school graduation	17 (68%)	16 (66.67%)		
High school graduation	3 (12%)	3 (12.50%)		
University/post-University degree	5 (20%)	5 (20.83%)		
Current Occupation, N (%)			χ^2^ (2, N = 49) = 0.17	0.29
Employed	19 (76%)	17 (70.83%)		
Not employed/Retired	6 (24%)	7 (58.33%)		

**Table 2:** Comparisons of psychological aspects between the Pilates group and the control group.

	Pilates Group (n = 25)		Control Group (n = 24)		t (48)	*p*	Cohen’s D
	M	SD	M	SD			
Symptom Questionnaire							
Anxiety	6.16	3.87	5.29	3.96	0.78	0.22	0.22
Depression	4.36	3.17	4.96	3.71	−0.61	0.27	−0.17
Somatization	6.64	4.08	8.58	4.9	−1.5	0.07	−0.43
Hostility	3.8	3.66	3.08	2.8	0.77	0.22	0.22
P Stress Questionnaire							
Sense of Responsibility	8	2.77	8.17	2.49	−0.22	0.41	−0.06
Vigor	2.16	1.95	1.63	1.76	1.01	0.16	0.29
Stress Disorder	4.16	1.31	4.5	1.32	−0.9	0.19	−0.26
Precision and Punctuality	4.6	1.63	5	1.52	−0.88	0.19	−0.25
Spare Time	2.08	1.88	2.42	2.21	−0.64	0.26	−0.18
Hyperactivity	5.8	0.91	5.71	1.04	−0.33	0.37	0.09
Total score	29.4	7.09	30.17	5.45	−0.42	0.34	−0.12

Legend: M = mean; SD = standard deviation.

**Table 3:** Repeated measures ANOVA for the Symptom Questionnaire scales with group, time, and time × group effects.

	Pilates Group				Control Group				Effects	
	(n = 25)				(n = 24)					
	T0	CI95%	T1	CI95%	T0	CI95%	T1	CI95%	F	η2
	(n = 24)		(n = 24)		(n = 25)		(n = 25)			
	M ± SD	LL-UL	M ± SD	LL-UL	M ± SD	LL-UL	M ± SD	LL-UL		
Anxiety	6.16 ± 3.87	4.58–7.73	3.92 ± 3.88	2.05–5.79	5.29 ± 3.96	3.68–6.70	8.21 ± 5.32	6.30–10.1	17.38 ***	0.27
Depression	4.36 ± 3.17	2.97–5.75	3.48 ± 3.24	1.86–5.10	4.96 ± 3.71	3.54–6.37	6.38 ± 4.72	4.72–8.03	5.44 *	0.1
Somatization	6.64 ± 4.08	4.83–8.45	4.48 ± 4.51	2.47–6.49	8.58 ± 4.90	6.74–10.43	10.33 ± 5.47	8.28–12.39	11.25 **	0.19
Hostility	3.80 ± 3.66	2.48–5.11	3.24 ± 3.46	1.89–4.59	3.08 ± 2.80	1.74–4.42	3.46 ± 3.24	2.08–4.83	0.83	0.02

Legend: M = mean; SD = standard deviation; * = *p* < 0.05; ** = *p* > 0.01; *** = *p* < 0.001.

**Table 4:** Repeated measures ANOVA for the P Stress Questionnaire scales with group, time, and time × group effects.

	Pilates Group				Control Group				Effects	
	(n = 25)				(n = 24)					
	T0	CI95%	T1	CI95%	T0	CI95%	T1	CI95%	F	η2
	(n = 24)		(n = 24)		(n = 25)		(n = 25)			
	M ± SD	LL-UL	M ± SD	LL-UL	M ± SD	LL-UL	M ± SD	LL-UL		
Sense of	8.00 ± 2.77	6.94–9.06	3.92 ± 3.88	6.54–8.19	8.17 ± 2.49	7.08–9.25	7.75 ± 2.72	6.59–8.11	0.05	0.001
Responsibility										
Vigor	2.16 ± 1.95	1.41–2.91	2.96 ± 2.43	2.05–3.87	1.63 ± 1.76	0.86–2.39	1.75 ± 2.09	0.82–2.68	1.57	0.03
Stress Disorder	4.16 ± 1.31	3.63–4.69	3.64 ± 1.38	3.05–4.23	4.50 ± 1.32	3.96–5.04	4.67 ± 1.55	4.06–5.27	4.27 *	0.08
Precision and Punctuality	4.60 ± 1.63	3.96–5.24	4.80 ± 1.66	4.11–5.43	5.00 ± 1.52	4.35–5.65	4.88 ± 1.45	4.24–5.52	1.2	0.03
Spare Time	2.08 ± 1.88	2.22–2.86	1.36 ± 1.68	0.66–2.06	2.42 ± 2.21	1.57–3.26	3.00 ± 1.82	2.28–3.72	8.56 **	0.15
Hyper-activity	5.80 ± 0.91	5.41–6.19	5.36 ± 1.15	4.94–5.78	5.71 ± 1.04	5.31–6.11	5.08 ± 0.93	4.65–5.51	0.55	0.01
Total score	29.40 ± 7.09	26.85–31.95	28.36 ± 7.92	25.78–31.24	30.17 ± 5.45	27.56–32.77	30.17 ± 6.27	27.23–33.11	1.06	0.02

Legend: M = mean; SD = standard deviation; * = *p* < 0.05; ** = *p* > 0.01.

## Data Availability

The dataset is available upon request from the authors. The raw data supporting the conclusions of this article will be made available by the authors on request.
